# Meteorological Table

**Published:** 1812-11

**Authors:** 


### METEOROLOGICAL TABLE.

### *From the 25th of September, to the 25th of October.*

| | D. | Therm. | Barom. | | Hygrom. *Dry.* | *Damp.* | Winds. | Atmos. Variation. |
|---|---|---|---|---|---|---|---|---|
| | 26 | 58 64 60 | $30^{1}$ | — | 3 12 6 | | W.. | C.. |
| ◐ | 27 | 61 65 63 | | | 2 4 5 | | W. | C. R. C. |
| | 28 | 63 64 58 | $29^{8}$ | — | | 1 4 6 | SW.. | C.. |
| | 29 | 57 60 59 | $29^{9}$ | 30 | | 4 7 1 | N.. | C... |
| | 30 | 60 62 61 | 30 | $29^{8}$ | | 5 8 3 | NW.. | C... |
| | 1 | 60 61 55 | $29^{8}$ | $^{9}$ | | 10 14 9 | W. | C... R.... F.. Thun |
| | 2 | 50 63 56 | — | $30^{1}$ | | 10 4 7 | N.. | C.. |
| | 3 | 56 64 56 | 30 | — | | 10 13 6 | SW.. | C.. |
| | 4 | 56 68 60 | | | | 9 4 1 | W... | F. — .... |
| ● | 5 | 55 66 60 | $29^{8}$ | $^{6}$ | | 4 8 — | SE. | C.. R... |
| | 6 | 62 66 61 | $29^{4}$ | $^{3}$ | | 10 8 4 | SW.. | F. R... |
| | 7 | 49 63 55 | $29^{4}$ | $^{3}$ | | 6 10 1 | S.. | F.. |
| | 8 | 48 64 53 | — | $29^{4}$ | | 12 7 1 | S. | R.. F. |
| | 9 | 49 56 54 | $29^{5}$ | — | | 10 2 5 | SW.. | R... F.. |
| | 10 | 50 55 51 | $29^{4}$ | — | | 9 9 14 | SE | R.. —... |
| | 11 | 54 56 52 | — | $29^{3}$ | | 7 4 8 | — | R. C.. R.. C.. |
| | 12 | 52 57 48 | | | | 2 14 8 | — | R.. C.. R.. C.. |
| ◑ | 13 | 50 56 50 | $29^{2}$ | 29 | | 3 2 6 | — | C.. F.. R.... |
| | 14 | 48 52 49 | $28^{9}$ | $29^{1}$ | | 10 9 8 | — | R.—.. F.. R.. F.. |
| | 15 | 49 54 48 | $29^{2}$ | $29^{5}$ | | 12 4 8 | W. | R. —.. F.. |
| | 16 | 52 56 48 | — | $29^{6}$ | | 3 6 5 | W.. | F... |
| | 17 | 48 58 53 | — | $29^{3}$ | | 7 6 10 | S.. | C.. |
| | 18 | 54 55 59 | — | $28^{9}$ | | 3 7 10 | SE... | F.. C.. R... |
| | 19 | 56 — 51 | — | $28^{7}$ | | 10 8 10 | W.. | R... —.... |
| ○ | 20 | 50 51 50 | $29^{1}$ | $^{3}$ | | 11 4 6 | NW... | F... |
| | 21 | 48 51 47 | $29^{6}$ | $^{9}$ | — 3 — | 1 — 6 | W... | F.. |
| | 22 | 54 56 51 | $29^{5}$ | $^{4}$ | | 10 4 7 | SW.. | C... |
| | 23 | 52 55 49 | $29^{5}$ | $29^{8}$ | | 6 1 6 | W... | F. R. F.. |
| | 24 | 46 54 50 | $29^{9}$ | — | | 8 2 3 | W.. | C. R. F.. |
| | 25 | 50 53 50 | $29^{6}$ | $^{5}$ | | 9 6 9 | SW.. | F. R.... F. |

Quantity of rain 3 inches $\frac{70}{100}$ from the 25th of September to the 25th of October. The wetness of this interval has been remarkable, and the range of the barometer has generally been low, and once on the 19th at $28^{7}$. On the 29th of September, a storm of thunder, which fell with violence on some parts of the metropolis; near the Tower, the lightning descended into the Thames, among the shipping, occasioning considerable alarm. With this thunder-storm the preceding dry weather broke up. October the 1st, thunder. It may be remarked, that the instruments intended to measure the pressure and humidity of the atmosphere, have corresponded with the actual state of the air with peculiar correctness.

In this interval, *Cholera* has been more prevalent than at any preceding period of the season; several individuals in the same family, have been attacked in a few hours. Some cases of Scarlatina have occurred, and one of what was called *Typhus*. The extension of the term *Typhus*, in a very general way, to cases of Fever, may warrant a more particular statement of this case. The patient, a man about 25, had lingered several days, neither well nor ill; after a fortnight of this state, he took to his bed. He had then dry but clean tongue, pulse below 100, not much increase of temperature, slight pain in the chest, with some cough. In three days from this time, the tongue became foul with a brown crust, and black sordes collected upon the teeth. He complained of his throat for two days, and which now, being inspected, was found deeply ulcerated, and in parts covered with gangrenous sloughs. The bowels, which before had been been constipated, suddenly fell into a dysenteric state, and large bloody dejections occurred six or seven times in 24 hours. The pulse during this period was never more than 110, the heat not much increased, no delirium, but the patient became obstinate, and almost stupid, except when required to take food or medicine, both of which for some days he refused with sullen pertinacity. From this alarming state, with spreading ulceration of the worst character in the throat, very frequent and copious discharges of dark blood per anum, and great debility, he gradually recovered. There are two reasons why we state this case: one, to question if it was a case of genuine typhus, propagating itself, by a contagion *sui generis;* the other, to state the probable good effects of *Cerivissia fermentum.* At the suggestion of an eminent physician, the patient was persuaded to take three tea-spoonfuls of yeast in a draught of the decoction Cinchonæ once in four hours. In 48 hours after adopting this remedy, the discharges from the bowels began to have a natural appearance, and were less frequent. It may be remarked, that, during the whole progress of the disease, the brain was free from morbid derangement; the throat, the chest, and the bowels, were the parts topically effected.

